# Doxorubicin cardiotoxicity and target cells: a broader perspective

**DOI:** 10.1186/s40959-016-0012-4

**Published:** 2016-03-03

**Authors:** Antonella De Angelis, Konrad Urbanek, Donato Cappetta, Elena Piegari, Loreta Pia Ciuffreda, Alessia Rivellino, Rosa Russo, Grazia Esposito, Francesco Rossi, Liberato Berrino

**Affiliations:** Department of Experimental Medicine, Section of Pharmacology, Second University of Naples, via Costantinopoli 16, 80138 Naples, Italy

**Keywords:** Doxorubicin cardiotoxicity, Myocardial homeostasis, Progenitor cells

## Abstract

The cardiotoxicity of doxorubicin is becoming an interdisciplinary point of interest given a growing population of cancer survivors. The complex and not completely understood pathogenesis of this complication makes difficult to design successful preventive or curative measures. Although cardiomyocyte has been considered a classical cellular target, other cells including various types of undifferentiated cells are involved in myocardial homeostasis. Such perspective may shed light on previously unrecognized aspects of cardiotoxicity and promote new experimental and clinical cardioprotective strategies. In this review, different cellular targets of doxorubicin are discussed with the focus on cardiac progenitor cells, oxidative stress, DNA damage, senescence and apoptosis all of which contribute to their compromised functional properties.

## Background

Anthracyclines, including doxorubicin (DOX), discovered nearly a half-century ago, are still a backbone of life-saving chemotherapy schemes [1]. Shortly after their introduction, the cardiovascular toxicity has been noticed and reported [2–5]. Although this class of drugs has been used and studied for decades, the pathogenesis of cardiotoxicity remains not completely understood. Its complex and still partially obscured nature makes difficult to design successful preventive or curative measures. Nowadays, given the accumulating population of cancer survivors that have been exposed to the treatment as children or adults, this problem is becoming an interdisciplinary point of interest. In this regard, relatively recent cardio-oncology initiative aims to respond to the growing needs to further clarify pathophysiology and to uniform the guidelines regarding diagnosis, prevention, management and follow-up of anthracycline cardiotoxicity [6].

Unfortunately, contrary to the common perception, the dimension of the problem is by no means small. One of the recent analyses reported the incidence of respectively subclinical and overt cardiotoxicity in 17.9 % and in 6.3 % of cancer patients treated with anthracyclines after 9 years follow-up [7]. In another study, scheduled echocardiography revealed left ventricular ejection fraction reduction in 9 % of adult patients within the first year after chemotherapy [8]. Moreover, with the use of alternative echocardiographic parameters as myocardial strain, high prevalence of cardiac dysfunction associated with anthracycline treatment has been reported, varying from nearly 30 % in adult survivors of childhood cancer to as much as 60 % in children [9, 10]. Taking to account the growing number of cancer survivors, that only in USA is predicted to reach about 19 million by 2024 [11], post-chemotherapy monitoring together with continued research on cardiotoxicity of anti-neoplastic drugs are warranted.

### Cardioprotective strategies

The first step to diminish the incidence of DOX cardiotoxicity is to use doses lower than 450 mg/m^2^, although it is becoming more and more apparent that while lowered cumulative dose causes a significant reduction of on-treatment events, no reduction of late-onset complications has been observed [12]. Therefore, there is no dose of DOX that can be considered absolutely safe.

Up to date, a series of strategies have been used in attempt to reduce or prevent deleterious effects that anthracyclines have on the heart. Proposed pharmacokinetics-based approaches consist of changing administration schedule by replacing bolus with slow infusion and switching from conventional to liposomal formulations [13]. In adults, continuous infusion that lowers peak concentration without affecting the dose may reduce cardiotoxicity without compromising anticancer activity [14]. However, the elevated costs of longer hospitalization and risk of infections have limited this method [15]. Additionally, in children, continuous infusion did not provide cardioprotective benefits [16]. Due to their peculiar distribution profile, uncoated or pegylated liposomal anthracyclines proved to be as effective as standard preparations but associate with minor cardiotoxic effects [17, 18]. Despite the availability of several formulations (i.e. liposomal DOX, pegylated liposomal DOX and liposomal daunorubicin), their cost and the lack of randomized trials in children with cancer [19], restricts their use to limited oncologic settings [20]. Another strategy is to utilize less cardiotoxic anthracycline derivatives, such as epirubicin or idarubicin, although their better safety profile is yet to be proven [21, 22]. However, it is often necessary to augment the dose to ensure the equivalent activity as that of DOX, thus increasing the risk of cardiotoxic events [23].

Other measures aim to prevent cardiotoxicity by interfering with molecular and cellular mechanisms altered by anthracycline. Dexrazoxane is the only approved cardioprotective agent used in patients exposed to anthracyclines [13], with a proven efficacy in childhood and adulthood [24, 25]. As an iron chelating agent, it interferes with iron-dependent redox reactions thereby decreasing reactive oxygen species (ROS) production and tissue damage acting as free-radical scavenger [26]. More recently, dexrazoxane was shown to inhibit DNA topoisomerase IIβ, thus preventing anthracycline from binding to the enzyme and consequent DNA double strand breaks [27]. A single, not confirmed report, which claimed a possible interaction of dexrazoxane with anti-tumour efficacy of anthracycline [28] together with another concern regarding the potential risk of a second malignancy in paediatric patients [24, 29, 30] led regulatory agencies to limit its clinical use [31]. However, this opinion may be worth to be re-evaluated [20, 32]. Other compounds with anti-oxidant properties, such as probucol, vitamin E, L-carnitine, coenzyme Q, glutatione and N-acetylcystein were tested in experimental and clinical settings with inconclusive findings [13, 33].

At present, there are no specific clinical practice guidelines and the treatment, as for all patients with heart failure, includes a combination of β-blockers, ACE-inhibitors, angiotensin receptor blockers, diuretics, nitrates, and hydralazine [34] and, for end-stage failure, heart transplantation. Among those, ACE-inhibitors and β-blockers showed significant cardiac protection in patients under anthracycline treatment [8, 35, 36]. Since the whole class does not share this effect, their action is probably not related to adrenoceptor blockage. In fact, carvedilol diminishes ROS formation in DOX-treated cardiomyocytes [37], whereas nebivolol prevents generation of peroxynitrite and NO synthase uncoupling [38].

Overall, available cardioprotective measures that operate directly or indirectly at the most accepted upstream phenomenon of ROS generation and oxidative stress [39] has not solved the clinical problem. Additional studies are necessary to develop alternative strategies for the heterogeneous patient population that carry for the rest of their lives the burden of higher risk of heart failure.

### Cellular targets

The myriad of studies aiming to explore cellular and molecular phenomena that may be clinically relevant has been focused on a cardiomyocyte and were extensively reviewed elsewhere [40, 41]. However, although cardiomyocyte has been considered a classical cellular target, other cell types such as cardiac fibroblasts, endothelial cells and vascular smooth muscle cells are also present (and are numerically prevalent) within the myocardium. Moreover, a notion that various types of undifferentiated cells can also be involved in the homeostasis of cardiac tissue certainly adds a new level of complexity to the understanding of myocardial biology. Because these elements dynamically interact in order to respond to changes in homeostatic needs and pathological stimuli, the structural and functional relationship between different cellular components cannot be dismissed. These considerations may offer the possibility to study previously unrecognized aspects of cardiotoxicity [42].

### Cardiac progenitor cells

The adult heart contains a population of primitive cells that in normal conditions contribute to tissue homeostasis, while in pathological states mediate myocardial regeneration [43–46]. Adult cardiac progenitor cells (CPCs) express c-kit, are self-renewing, clonogenic and multipotent, give rise to cardiomyocytes, smooth muscle cells and endothelial cells. CPC involvement has been documented in aging and in several pathological conditions also in humans [47–52], indicating this cells as a pathophysiological target. Furthermore, senescence of stem cell population contributes to the onset and progression of heart failure [47–49, 51–54]. In this view, relatively recent studies have shown that cardiotoxicity of the anthracycline is not restricted to cardiomyocytes but affects also resident CPCs, proposing an additional mechanism underlining the pathophysiology of DOX-induced cardiomyopathy [55–58]. In a model of anthracycline-induced heart failure, DOX inhibited CPC proliferation, that in combination with the accumulation of oxidative DNA damage, growth arrest, cellular senescence and apoptosis led to an almost complete depletion of the CPC pool. The lack of activation of CPCs interfered with the turnover of cardiomyocytes in the presence of myocyte death and senescence [56].

The clinical relevance of these animal findings was established by the study performed on hearts obtained at autopsy from oncologic patients who died of heart failure developed after treatment with chemotherapeutic regimens including anthracyclines. The hearts of patients with anthracycline cardiomyopathy contained higher fraction of senescent human CPCs (hCPCs) when compared with age-matched controls that died from non-cardiovascular causes [57]. The senescence marker p16^INK4a^ was present in the vast majority of hCPCs, exceeding the values reported for chronologic aging and other cardiomyopathies [47, 48, 54]. These observations were complemented by the in vitro tests showing that isolated hCPCs were sensitive to DOX, and their survival, growth and function were negatively affected. DNA damage in hCPCs, shown by the expression of a phosphorylated form of histone H2 (γH2AX), and the accumulation of senescent cells could therefore affect cardiac homeostasis increasing susceptibility to the myocardial damage also in the human heart.

### DOX and functional properties of CPCs

To fulfil their role, the viable progenitor cells need to reach the area of injury and give rise to differentiated progeny capable to repair the damage. IGF-1/IGF-1R and HGF/c-Met systems are determinants of CPC function favouring CPC-mediated myocardial regeneration [59, 60]. Stimulation of IGF-1R activates mitogenic and antiapoptotic effects [59, 61, 62] while c-Met is the receptor for HGF, a cytokine that stimulates cell migration to the sites of injury [62, 63]. DOX reduces the expression of IGF-1R and c-Met in hCPCs and this negative effect persists with time [57]. Although DOX does not abolish differentiation capacity of hCPCs, the majority of committed progeny deriving from DOX-treated progenitors prematurely inherited a senescent phenotype. In addition to CPC death and senescence, DOX negative interference with growth factor systems that regulate cardiac repair, may further aggravate the inadequate response of the human heart to stress. The functional inferiority of hCPCs exposed to DOX was confirmed *in vivo* in an animal model of anthracycline cardiomyopathy. In contrast to experimental therapy with healthy cells, the use of DOX-treated hCPCs did not lead to structural and functional recovery and the survival benefits were not observed [58].

### CPCs and late onset cardiotoxicity

The unresolved enigma however, is that the time elapsed between therapy with anthracyclines and onset of cardiac complications varies from months to years or even decades. It is possible that minor noxious events, that in the healthy person would not have severe consequences, can trigger pathophysiological cascade in subjects treated with anthracyclines. Pre-existing conditions or co-morbidities that develop during the post-chemotherapy period can additionally increase the risk of the cardiovascular sequel. An intriguing working hypothesis is that therapy with anthracyclines can leave a specific cellular “signature” to the heart that persists with time and reveals itself after the latent and asymptomatic period with the devastating outcome. In this scenario, a long-lasting damage of a quiescent progenitor cell can serve as a “carrier” of this information. This concept was tested in the study of late-onset cardiotoxicity in mice injected with DOX shortly after birth [55]. In adult age, no difference in cardiac function was observed and the hearts from DOX-treated mice appeared morphologically normal without degenerative changes suggestive of DOX-mediated toxicity. However, the juvenile DOX exposure reduced coronary flow, vessel branching and capillary density, suggesting that juvenile DOX exposure might affect vascular development. Interestingly, hearts of adult mice injected with DOX in juvenile age are more sensitive to stress (i.e. exercise or myocardial infarction, MI) and displayed sign of late-onset cardiotoxicity. Moreover, the post-MI survival of DOX-exposed mice was lower and these animals had a reduced neovascularization suggesting that DOX treatment puts the adult heart at higher risk for ischemic injury. Although MI was associated with the migration of progenitor cells into the damaged area, there were significantly fewer CPCs in the border zone of animals that had been exposed to DOX as pups. This study also suggested that juvenile DOX exposure affects differentiation of CPCs. Of note, DOX-exposed pups had significantly fewer CPCs, reinforcing the hypothesis that DOX might be harmful to these cells. CPC growth and telomerase activity were reduced while cell cycle inhibitor p16^INK4a^ was upregulated. Therefore, juvenile exposure even to low dose of DOX induced senescence and might have permanently reduced the number of resident CPCs.

The possibility that cellular senescence can represent an early event also in the human heart is supported by the presence of high fraction of p16^INK4a^-positive hCPCs in myocardium of anthracycline-treated patients with normal cardiac function that died of other complications during chemotherapy [57]. Because the early and late cellular adaptations that occur after treatment may differ, the evolution of the cellular and molecular effects of clinically relevant concentrations of DOX on hCPCs was studied in vitro. Early after exposure, DOX reduced hCPC viability, induced significant level of apoptosis and increased the expression of proteins involved in the DNA damage response such as phospho-ATM^Ser1981^ kinase, γH2AX and phospho-p53^Ser15^ [57]. The activation of ATM coupled with the increase of γH2AX indicates DNA damage while the phosphorylation of p53 can activate apoptotic or cellular senescence pathways [64]. In DOX-treated hCPCs, the early increase in p53 phosphorylation triggered the late activation of apoptotic while the expression of p16^INK4a^ began to rise in parallel with the increased activity of SA-β-gal [57]. Similar cellular events can occur in the hearts of patients during or immediately after DOX administration. However, after removal of DOX, the rate of apoptotic death of hCPCs and the expression of proteins involved in DNA damage response (phospho-p53^Ser15^, phospho-ATM^Ser1981^) returned to baseline. In the absence of other pro-senescence stimuli a cell could potentially resume its proliferative capacity [65] but this was not a case. The very high fraction of senescent cells indicated that hCPCs entered the irreversible phase of growth arrest after permanent activation of p16^INK4a^-Rb pathway. In this scenario, a transient activation of p53 initiates the senescence response while p16^INK4a^ operates to maintain this state representing a delayed cellular response [64, 66].

Although the reported phenomena *per se* may not directly lead to heart failure, they can make the myocardium of an apparently healthy person more vulnerable. The late onset of cardiomyopathy in patients, who already have sustained subclinical cardiac damage as a result of DOX chemotherapy, could be attributed to an additional pathological or physiological stress like ischemia, acute viral infection, exercise, pregnancy, or the increase of body mass in children during normal growth. These factors can transform the silent myopathy into overt heart failure [10, 67–69].

### Other cardiac cells

#### Cardiac fibroblasts

Accumulation of fibrotic tissue is one of the features of DOX-cardiomyopathy triggered by the necrotic damage and its pro-inflammatory load, but also by the excessive ROS generation. The latter has been recognized as a strong stimulator of pathological collagen production as well as activator of transforming growth factor-β (TGF-β), a cytokine that has been associated to fibro-inflammatory signaling [70–72]. In our model, DOX determined significant increases of TGF-β and phospho-SMAD3 along with an increment of collagen deposition, and enhanced phenotypic transformation of fibroblasts to myofibroblasts, cells that express contractile proteins and secrete pro-fibrotic factors [73]. Interestingly, cardiac fibroblasts isolated from DOX-treated rats had elevated levels of TGF-β and an increased phospho-SMAD3^Ser423/425^/SMAD3 ratio. The expression of fibroblast activate protein 1α and α-SMA, markers of activated fibroblasts was also augmented. Additionally, DOX significantly upregulated two of the main components of the extracellular matrix, collagen type 1 and fibronectin [73]. Therefore, these results show that in vivo exposure to DOX generates pro-fibrotic population of activated cardiac fibroblasts. Surprisingly, the activated, pro-fibrotic phenotype of cardiac fibroblasts was preserved *in vitro* even after several passages, suggesting once again that the cell that has been stressed with DOX retains the “pharmacological signature” and transmits this state to the progeny. This intriguing phenomenon requires specific future investigations.

Finally, because fibroblasts play a role as supporting cells for CPCs within the niches, the potential damage produced by DOX can result in the derangement of the cardiac niche [74]. The biological role of myofibroblast-endogenous stem cell interactions, in terms of cardiac fibrosis, remains unclear, although several studies suggest that also progenitor cells may influence extracellular matrix composition in a paracrine manner [75].

### Vascular cells

Since both experimental and clinical studies reported that anthracycline toxicity may be also linked to the deleterious effects of these drugs on the endothelium [76], a brief examination of smooth muscle cells (SMCs) and endothelial cells has to be done. In particular, in vitro and ex-vivo studies evaluated SMCs responses to drug exposure. DOX-treated vascular SMCs were arrested in the G2/M phase of the cell cycle and a series of classical markers of cellular damage and senescence (i.e. SA-β-gal activity, DNA damage foci, changed morphology, and increased superoxide production) were observed [77]. Instead, organ-culture vascular tissue was used to examine the effects of DOX on the morphology and functions of SMCs. Interestingly, treatment with DOX decreased the α adrenoceptor protein and noradrenaline-induced contraction. The mediation of ROS was demonstrated by the partial restoration of α adrenoceptor expression and vessel contraction in presence of SOD [78]. Moreover, an in vitro study conducted on endothelial cells documented as both DOX and daunorubicin induced ROS-dependent cytotoxic effects, although with different potency due to their relative cellular accumulation [79].

### Extracardiac cells with progenitor properties

As discussed above, the inhibition of the progenitor cell-mediated self-repairing potential of the heart is considered one of the pathogenetic mechanisms of DOX-induced cardiomyopathy. Apart cardiac progenitors, also different cell types of extra-cardiac origin, such as bone marrow, could be taken into account to better understand pathophysiology of the heart after injury and to extend the knowledge about the role of stem cells in DOX-induced cardiomyopathy.

### Endothelial progenitor cells

Bone marrow-derived endothelial progenitor cells (EPCs) can be mobilized to the peripheral circulation thus contributing to post-natal angiogenesis and vasculogenesis [80]. Recruitment of these cells was found to attenuate tissue damage in models of myocardial ischemia and anthracycline cardiomyopathy [81–83]. Also in patients, number of circulating EPCs has been seen to be predictive of cardiovascular diseases and may represent a better predictor of vascular reactivity than conventional cardiovascular risk factors [84]. Interestingly, in vitro studies reported dose-dependent DOX toxicity on EPCs in which apoptosis was induced by high doses while low doses of DOX caused a premature senescence phenotype. Sub-cytotoxic dose of DOX produces ROS with the result of alteration in the expression of several proteins that regulate cell cycle, cytoskeletal and cellular architecture [85, 86]. Oxidative stress may trigger the p16^INK4a^-pRb-dependent senescence involving p38 signalling [87]. EPCs exposed to sub-apoptotic doses of DOX undergo the activation of MAPKs p38 and JNK signalling, whose balance regulates senescence and apoptosis [86, 88, 89]. p38 and JNK pathways antagonistically control cellular senescence, p16^INK4a^ expression and cytoskeletal organization in EPCs treated with DOX [86]. It is therefore possible that also in EPCs, ROS accumulation, induction of p16^INK4a^ together with telomere dysfunction, suggested by the down regulation of TRF2, jointly contribute to DOX-induced senescence of this progenitor cell class leading to the failure of EPC-mediated regenerative processes. Of note, in an experimental model, DOX-induced nephropathy was associated with development of EPC senescence and incompetence that hampered their engraftment into the kidney, supporting the hypothesis that progenitor cells injured by DOX are, in part, responsible for the progression of the disease [90].

### Bone marrow cells

In physiologic conditions, the trafficking of bone marrow cells (BMCs) to other organs is limited. However, after tissue damage, this process is amplified and a massive number of progenitor cells are released into the peripheral blood with the involvement of several mediators, such as granulocyte-colony stimulating factor (G-CSF), stromal cell-derived factor-1 and vascular endothelial growth factor [91, 92]. It has been shown that also human heart can be the site of homing of BMCs and that cardiomyocytes and coronary vascular cells can be formed de novo in the adult life [93, 94]. Experimental models confirmed the possibility that BMCs can translocate from the marrow to the infarcted myocardium and contribute to the repair process [95]. Although a significant amount of clinical work has been published regarding the effect of the BMCs mobilization on the heart after an acute ischemic injury, inconclusive results have been achieved with divergent evidence reported about the possibility that G-CSF-activated BMCs may improve left ventricular ejection fraction or attenuate ventricular remodelling [96–98]. Of note, also in a mouse model of DOX cardiotoxicity, G-CSF has been used to mobilize BMCs for evaluation of their migration capacity towards the myocardium and their possible role in attenuating the cardiac dysfunction. G-CSF enhanced the migration of BMCs into the heart, attenuated cardiotoxicity and improved survival. Moreover, green fluorescent protein-labelled BMCs observed were structurally integrated in the myocardium and acquired a myocyte-like phenotype [99]. Although the clinical importance of the bone marrow mesenchymal stem cell (MSC)-driven response to the cardiac damage is unknown, there is a consensus that MSCs can contribute directly and indirectly [100–102] to the repair of the damaged myocardium, and the clinical trials with MSCs and chronic ischemic heart failure are ongoing [103]. It should be pointed out that the bone marrow, although provided with an active population of stem cells, does (like any other system) fail under prolonged toxic injury. In this regard, given the cytotoxic properties of DOX, bone marrow toxicity represents the limiting factor during the treatment. When chemotherapeutic regimens disregard regular time intervals to allow the recovery of the hematopoietic system, bone marrow failure leads to life-threating intractable septic and haemorrhagic complications [104]. Therefore, it is reasonable to assume that bone marrow-derived stem cells “primed” with DOX undergo alterations that can interfere with their function also in other organs. In fact, it has been shown that DOX is toxic for MSCs [105, 106]. In the view of potential clinical interest for MSCs, this observations need further investigation.

## Conclusions

Although only a few studies addressing the role of cells other than cardiomyocytes are available, it is reasonable to assume that molecular events that occur at level of different cellular compartments contribute to development and progression of DOX cardiomyopathy. Additionally, independently from the molecular mechanisms through which DOX induces cellular damage, oxidative stress, DNA damage, senescence and apoptosis all happen and have an impact on myocardial homeostasis, although their relative weight can differ. Therefore, to facilitate the comprehension of the sequence of events emerging from the complex interaction of different forms of molecular stress, more detailed studies should address not only the role of each cell type but also the possibly different susceptibility of each cell.

Adverse effects present long after the clearance of the drug and its metabolites remain another unresolved issue. A challenging hypothesis yet to be tested involves a phenomenon of biological memory, described as a persistent cellular response to a transitory stimulus. Cellular memory of past stimuli maintains cell identity even if the signal was experienced only once [107]. In addition to normal development, such phenomenon has also been described in diabetes when a cell “remembers” the onset of a hyperglycaemic peak [108]. In this view, an anthracycline can be considered as a pharmacological stressor that leaves its molecular signature in different cellular components probably through specific epigenetic changes. Interestingly, ROS have been proposed as one of the molecular keepers of metabolic memory [109] and take an undisputable part in cardiovascular diseases by modulating numerous cellular processes like cellular migration, proliferation and hypertrophy, angiogenesis, apoptosis and senescence, all of which contribute to cardiotoxicity. Therefore, although advanced knowledge of biological and cellular events implicated in the response of the heart to anti-cancer therapy is a fundamental tool, the further dissection of events should not drive our attention away from a central role of a ROS-driven processes that sustain an on-going damage and create a state of susceptibility. Thus, a broader cellular view presented here combined with ROS-modifying approaches could be a useful platform for new experimental and clinical cardioprotective strategies.

### Ethics approval and consent to participate

Not applicable.

### Consent for publication

Not applicable.

## Abbreviation

BMCs: bone marrow cells
CPCs: cardiac progenitor cells
DOX: doxorubicin
EPCs: endothelial progenitor cells
G-CSF: granulocyte-colony stimulating factor
hCPCs: human cardiac progenitor cells
MI: myocardial infarction
MSCs: mesenchymal stem cells
ROS: reactive oxygen species
SMCs: smooth muscle cells
TGF-β: transforming growth factor-β
γH2AX: phospho-histone H2
